# RNA sequencing reveals the potential mechanism of exercise preconditioning for cerebral ischemia reperfusion injury in rats

**DOI:** 10.1002/brb3.3608

**Published:** 2024-07-02

**Authors:** Yan Wu, Hui Yang, Feifeng Chen, Baohua Li, Xiangbo Meng

**Affiliations:** ^1^ Department of Rehabilitation Medicine Hangzhou First People's Hospital Hangzhou Zhejiang China; ^2^ Department of Neurology Hangzhou First People's Hospital Hangzhou Zhejiang China; ^3^ Department of Rehabilitation Medicine The Affiliated Hospital of Hangzhou Normal University Hangzhou Zhejiang China

**Keywords:** cerebral ischemia reperfusion injury, exercise preconditioning, RNA sequencing, TIMP1/HIF‐1 pathway

## Abstract

**Introduction:**

Cerebral ischemia reperfusion injury (CIRI) often leads to deleterious complications after stroke patients receive reperfusion therapy. Exercise preconditioning (EP) has been reported to facilitate brain function recovery. We aim to explore the specific mechanism of EP in CIRI.

**Methods:**

Sprague‐Dawley rats were randomized into Sham, middle cerebral artery occlusion (MCAO), and EP groups (*n* = 11). The rats in the EP group received adaptive training for 3 days (10 m/min, 20 min/day, with a 0° incline) and formal training for 3 weeks (6 days/week, 25 m/min, 30 min/day, with a 0° incline). Then, rats underwent MCAO surgery to establish CIRI models. After 48 h, neurological deficits and cerebral infarction of the rats were measured. Neuronal death and apoptosis in the cerebral cortices were detected. Furthermore, RNA sequencing was conducted to investigate the specific mechanism of EP on CIRI, and qPCR and Western blotting were further applied to confirm RNA sequencing results.

**Results:**

EP improved neurological deficit scores and reduced cerebral infarction in MCAO rats. Additionally, pre‐ischemic exercise also alleviated neuronal death and apoptosis of the cerebral cortices in MCAO rats. Importantly, 17 differentially expressed genes (DEGs) were identified through RNA sequencing, and these DEGs were mainly enriched in the HIF‐1 pathway, cellular senescence, proteoglycans in cancer, and so on. qPCR and Western blotting further confirmed that EP could suppress TIMP1, SOCS3, ANGPTL4, CDO1, and SERPINE1 expressions in MCAO rats.

**Conclusion:**

EP can improve CIRI in vivo, the mechanism may relate to TIMP1 expression and HIF‐1 pathway, which provided novel targets for CIRI treatment.

## Graphical abstract

The role and mechanism of exercise preconditioning on cerebral ischemia.

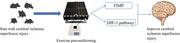

## 1 INTRODUCTION

Ischemic stroke accounts for about 70% of stroke cases worldwide, which poses a severe health hazard to humans due to its high mortality and disability rates as well as the occurrence of numerous serious complications Wang et al., 2022). Revascularization and thrombolysis, which are used to recover the blood supply for infarct area, are the primary treatments used in clinics for patients with cerebral ischemia (CI) (Yan et al., 2020). Nonetheless, the therapeutic window time for thrombolysis is very short, and its efficacy and safety are strictly time dependent (Yu et al., 2020). Although reperfusion usually can effectively restore the blood flow to ischemic regions, such a technique may also cause tissue damage, dysfunction, and even deterioration (He et al., 2019). This phenomenon is called CI reperfusion injury (CIRI) in clinic.

Exercise in daily life not only benefits the nervous system but also improves brain functions by enhancing learning, memory, cognition, and plasticity (Vilela et al., 2020). Exercise preconditioning (EP) is defined as the repetitive whole‐body exercise performed before ischemia, which may induce ischemic tolerance against numerous secondary brain damages resulting from CIRI, thereby effectively improving brain damage (Zhang et al., 2022). Relative to other preconditioning methods, EP is more favorable as its easy, noninvasive, low‐cost but high efficiency. Published reports have shown that exercise could decrease the risk of stroke recurrence (Lin et al., 2021) and promote neuroprotection in CIRI (Dornbos & Ding, 2012); however, the precise mechanisms of EP on CIRI are still unclear.

Transcriptome sequencing analysis can provide a comprehensive understanding of the exact molecular mechanisms and biological processes that participate in disorders by studying the information of genes (Banerjee et al., 2023). Transcriptome sequencing analysis is important in disease diagnoses and novel drug development. At present, RNA sequencing technology is the preferred method for transcriptome sequencing, which has been utilized in multiple fields such as clinical medicine, biology as well as pharmaceutical research (Shi et al., 2019). RNA sequencing conducted by Liu, Liu et al. (2021) has reported that β‐caryophyllene may exert neuroprotective functions in CIRI rats by reducing MMP9, TIMP1, and STAT3 expressions. In addition, a study also reported that the HIF‐1 pathway was activated in middle cerebral artery occlusion (MCAO) rats (Yu et al., 2021). A previous study has confirmed that exercise, both aerobic treadmill training and weighted stair‐climbing training, can decrease TIMP1 expression in insulin‐resistant mice (Wang, 2019). Similarly, exercise has also been found to ameliorate various diseases, including myocardial infarction (Song & Tian, 2023) and pregnancy complications in mice (Alizadeh Pahlavani, 2022), by inhibiting the HIF‐1 pathway. However, there is currently no relevant research using RNA sequencing to explore the potential mechanisms of EP in CIRI.

Thus, in this study, rats received EP before MCAO surgery, then, the effects of EP on CIRI were detected. Thereafter, RNA sequencing technology was employed to comprehensively explore the underlying mechanism of EP in CIRI, thereby providing a scientific foundation for CIRI prevention.

## 2 MATERIALS AND METHODS

### 2.1 Ethics

All animal‐related procedures were adhered to the Institutional Animal Care and Use Committee. Ethics approval was obtained from the Animal Experimentation Ethics Committee of Zhejiang Eyong Pharmaceutical Research and Development Center (Certificate No. SYXK (Zhe) 2021‐0033).

### 2.2 Animals and grouping

Thirty‐three male Sprague‐Dawley rats (8 weeks, 250–300 g) from Shanghai Jihui Laboratory Animal Care Co., Ltd (license No. SCXK (Hu) 2017‐0012) were randomly divided into three groups (*n* = 11/group): Sham, MCAO, and EP groups. The rats were housed in animal rooms with 50% humidity and 23 ± 1°C temperature‐controlled air‐conditioning, under 12 versus 12 h light‐dark cycles.

### 2.3 Exercise intervention

The exercise intervention protocols for the current research were developed based on previous studies (Zhu et al., 2016).

All rats received adaptive treadmill training for 3 consecutive days before the formal training. The adaptive training protocol was composed of 20 min/day walking on a treadmill at a speed of 10 m/min, with an incline of 0°. Following the completion of adaptive training, formal training began, that is, rats of the EP group were subjected to a 3‐week treadmill training that was conducted 6 days/week. The formal training protocol was set at running for 25 m/min, with a 0° incline, 30 min/day. In order to force the rat to exercise, an electric shock area was established at the back of the running platform. Rats refused to run would fall into the electric shock area and get an electric stimulus. The intensity of the shock stimulus was set at 1.5 mA. At the same time, rats in the Sham as well as MCAO groups were given unrestricted exercise for 3 weeks.

### 2.4 MCAO surgery

The approach described by Longa with slightly modified was used to induce the MCAO rat model (Longa et al., 1989). Before surgery, the rats from the MCAO and EP groups were fasted 12 h for food, but not water. Then, rats were anesthetized by 1.5%–2% isoflurane, after the righting reflex disappeared, 1.5% isoflurane was applied to maintain the anesthesia. Afterward, the skin around the neck was prepared, and an incision on the right of the midline was made. Next, the subcutaneous fascia, muscle as well as right acinus were bluntly dissected to uncover the surgical region. The right common carotid artery (CCA), internal carotid artery (ICA), and external carotid artery (ECA) were then exposed and separated. Subsequently, silk suture was applied to ligate the ICA and CCA temporarily. Moreover, the distal end of the ECA was ligated permanently, and a silicone‐coated nylon suture was gently inserted to the ICA from the ECA until felt slight resistance. Hereafter, we tied a slipknot around the CCA. After 1.5 h of blood flow interruption, the nylon suture was carefully withdrawn for blood flow restoration to induce reperfusion. During the surgery, rats’ body temperature was closely monitored and kept at 37 ± 0.5°C. For the rats from the Sham group, the surgical procedure was carried out in the same manner, except for the insertion of a nylon suture from the ECA into the ICA.

### 2.5 Neurological deficit evaluation

Neurological deficits of the rats were evaluated 48 h post CIRI by a scoring system, with scores ranging from 0 to 5, and the score criteria were described as followed to assess neurological deficits in experimental animals: 0 point, normal activity with no symptoms of neurological deficit; 1 point, light neurologic deficit, such as inability to stretch the contralateral forepaw fully; 2 points, medium neurologic deficit, including circling toward the contralateral side while crawling; 3 points, serious neurologic deficit, such as turning to the contralateral side while crawling; 4 points, losing consciousness and inability to crawl; 5 points, death.

### 2.6 Tissue collection

After completion of neurological deficit evaluation, animals were euthanized by inhaling an overdose of isoflurane. Three rats from each group were chosen for 2,3,5‐triphenyl tetrazolium chloride (TTC) staining. The leftover brains were rapidly dissected, and the cortical tissues were isolated for subsequent Nissl as well as TdT‐mediated dUTP nick end labeling (TUNEL) staining, with fixation in 4% paraformaldehyde (PFA) and dehydration prior to embedding in paraffin and sectioning at a thickness of 4 µm. The rest of the cerebral cortical tissues were kept at −80°C for further RNA sequencing, qPCR as well as Western blotting.

### 2.7 Nissl staining

Neuronal death in the cerebral cortices was examined through Nissl staining. In short, the tissue slices were deparaffinized with xylene and then hydrated via ethanol. Following this, the slices were stained by 1% Toluidine blue staining (71041284, Beijing OKA Biological Technology Co., Ltd) for 20–40 min. At last, a light microscope was employed to observe the morphological changes of neurons. In addition, the mortality of neurons in cortical tissues was assessed by ImageJ.

### 2.8 TTC staining

Cerebral infarction of the rats was detected by TTC staining. First, phosphate buffer was utilized to prepare TTC solution at 37°C in a darkened environment. The brain tissues of the rats from each group were quickly removed and cut into coronal slices with 2 mm thick. Then, the brain tissues were incubated in 1% TTC solution, followed by fixation with 4% PFA overnight. At last, the infarct size of the brain was photographed and quantified by ImageJ.

### 2.9 TUNEL staining

TUNEL staining was utilized to evaluate apoptosis in resected cerebral cortical tissues. Briefly, processed paraffin slices were dewaxed using xylene and rehydrated in ethanol. Proteinase K was then applied to treat the tissue sections at 37°C for 30 min. Following washing, the samples were incubated with TUNEL solution (C1090, Biyuntian) in the dark for 2 h. Subsequently, the samples were stained with 4', 6‐diamidino‐2‐phenylindole (DAPI) at room temperature. Finally, a fluorescence microscope was used to view and take photos of the sections.

### 2.10 RNA sequencing

For RNA sequencing, five cerebral cortical tissues were randomly chosen from Sham, MCAO, and EP groups. The tissues were treated with liquid nitrogen before being sent to Azenta Life Sciences for RNA sequencing. The Illumina/MGI sequencing platform was used for the experiment, and the library construction was done using the Illumina Truseq RNA sample prep kit method. Cutadapt (V1.9.1) was applied for removing the adapter and low‐quality sequences. Then, Hisat2 software (V2.0.1) was used to align the clean data to the reference genome. The differentially expressed genes (DEGs) of the cerebral cortical tissues among Sham, MCAO, and EP groups were analyzed using the DESeq2 Bioconductor package. The threshold for DEGs was set as false discovery rate (FDR) less than 0.05 and fold change greater than 2. To further understand the DEGs, Gene ontology (GO) as well as Kyoto Encyclopedia of Genes and Genomes (KEGG) databases were utilized for enrichment analysis.

### 2.11 qPCR

The expressions of TIMP1, SOCS3, ANGPTL4, CDO1, and SERPINE1 mRNA in cerebral cortical tissues were assessed by qPCR. The total RNA of the cerebral cortical tissues was isolated using the EZ‐10 total RNA small amount extraction kit (B618583‐0100, Sangon Biotech), following which cDNA was synthesized with reverse transcription kits (CW2569, CWBIO). The synthesized cDNA was quantified by qPCR using SYBR Green qPCR kits (11201ES08, Yeasen). β‐actin served as an internal reference, and the relative expression levels of TIMP1, SOCS3, ANGPTL4, CDO1, and SERPINE1 mRNA were calculated using the 2^−ΔΔ^ *^Ct^* method. The primer sequences used in this study were presented in Table 1.

**TABLE 1:** Primer sequence of the genes for qPCR analysis.

Gene	Forward primer	Reverse primer
Rat TIMP1	AGCTTTCTGCAACTCGGACC	TCGAGACCCCAAGGTATTGC
Rat SOCS3	GCAGGAGAGCGGATTCTACT	TGGTCCAGGAACTCCCGAAT
Rat ANGPTL4	GAAATGAACTTGCTGGCTCAC	TAGTGGATAGTAGCGGCCCTT
Rat CDO1	GCCGACCTCATCCGAATCTT	CCTGTTCGTTGGTCAAAGGC
Rat SERPINE1	TTTGGGAAAGGGTTCGCTTC	GGCTGAGACTAGAATGGCTGT
Rat β‐actin	AAGGCCAACCGTGAAAAGAT	GCTCGAAGTCTAGGGCACA

### 2.12 Western blotting

The total protein of cerebral cortical tissues was extracted by radioimmunoprecipitation buffer (P0013B, Biyuntian), separated with 10% SDS‐polyacrylamide gel electrophoresis and transferred to polyvinylidene difluoride membranes. After hindering with 5% non‐fat milk, the membranes were incubated overnight at 4°C with primary antibodies against TIMP1 (AF7007), SOCS3 (DF6133), ANGPTL4 (DF6751), CDO1 (DF10174), SERPINE1 (BF0086), and β‐actin (81115‐1‐RR). Then, the membranes were probed with horseradish peroxidase (HRP)s (‐conjugated secondary antibody (1:6000, 7074, CST) for 1 h. The bands were visualized using chemiluminescence and quantified by ImageJ software. β‐actin served as an internal control. All primary antibodies were purchased from Affinity and used at a dilution of 1:1000, except for β‐actin, which was used at a dilution of 1:10,000.

### 2.13 Statistical analysis

The analysis of the study was conducted by SPSS V19.0, and the calculated results were shown as means ± SD. Multiple group comparisons were examined using one‐way analysis of variance and Tukey tests. In addition, the Kruskal–Wallis *H* test was utilized when the variances were not homogeneous. A statistically significant result was defined as *p* < .05 in all cases.

## 3 RESULTS

### 3.1 EP improved the neurological deficit scores in MCAO rats

**FIGURE 1:**
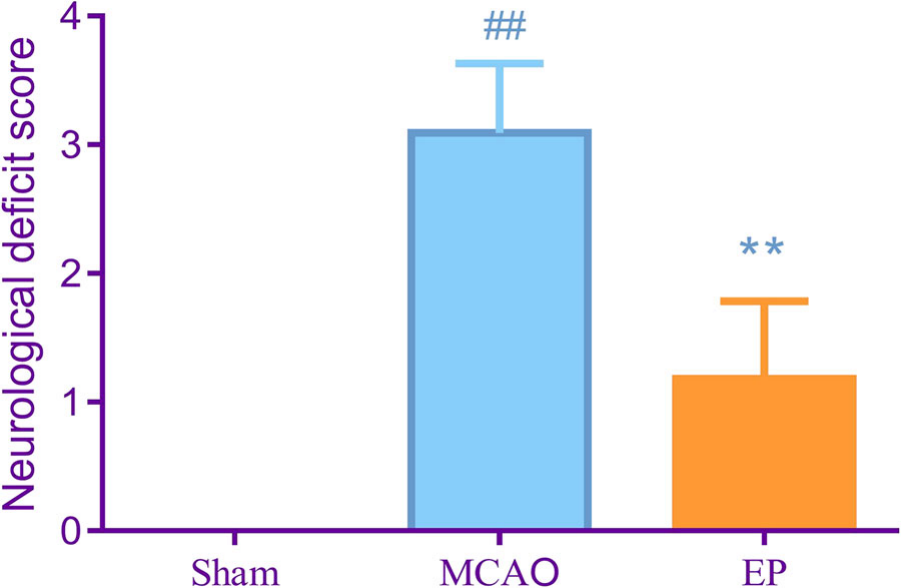
EP alleviated the neurological deficit scores in MCAO rats. The neurological deficit scores in each group were assessed 48 h following CIRI. ^#^ *p* < .05, ^##^ *p* < .01 versus Sham. *p < .05, **p < .01 versus EP. Results were presented as mean ± SD. *n* = 11. CIRI, cerebral ischemia reperfusion injury; EP, exercise preconditioning; MCAO, middle cerebral artery occlusion.

Neurological deficit scores were assessed 48 h following CIRI. As illustrated in Figure 1, relative to the Sham rats, rats from the MCAO groups displayed obvious neurological deficits (*p* < .01). However, EP notably reduced the neurological deficit scores 48 h after CIRI (*p* < .01).

### 3.2 EP alleviated the neuronal death of the brain in MCAO rats

**FIGURE 2:**
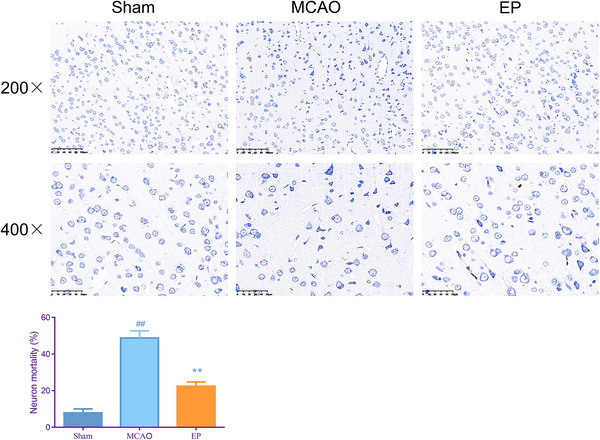
Exercise preconditioning (EP) alleviated neuronal death of the cerebral cortices in middle cerebral artery occlusion (MCAO) rats. At 48 h after cerebral ischemia reperfusion injury (CIRI), the neuronal death of the cerebral cortices in each group was measured by Nissl staining (magnification, 200×, scale bar = 100 µm; magnification, 400×; scale bar = 50 µm). ^#^ *p* < .05, ^##^ *p* < .01 versus Sham. *p < .05, **p < .01 versus EP. Results were presented as mean ± SD. *n* = 3.

To investigate whether EP can help alleviate MCAO‐caused neuronal death in the brain, we examined neuron morphology and mortality by Nissl staining. As exhibited in Figure 2, neuronal cells in the cerebral cortical tissues of the Sham group appeared light blue, with a relatively intact cellular structure. However, in the MCAO group, more neuronal cells in the cerebral cortical tissues were wrinkled and appeared dark blue. Compared to the MCAO group, there were fewer wrinkled neuronal cells in the brain tissues of rats in the EP group. In addition, we observed an increase in neuron mortality in the MCAO group (*p* < .01). Although pretreated with exercise, the number of dead neurons in the EP group was significantly downregulated (*p* < .01).

### 3.3 EP‐attenuated cerebral infarct volume in MCAO rats

**FIGURE 3:**
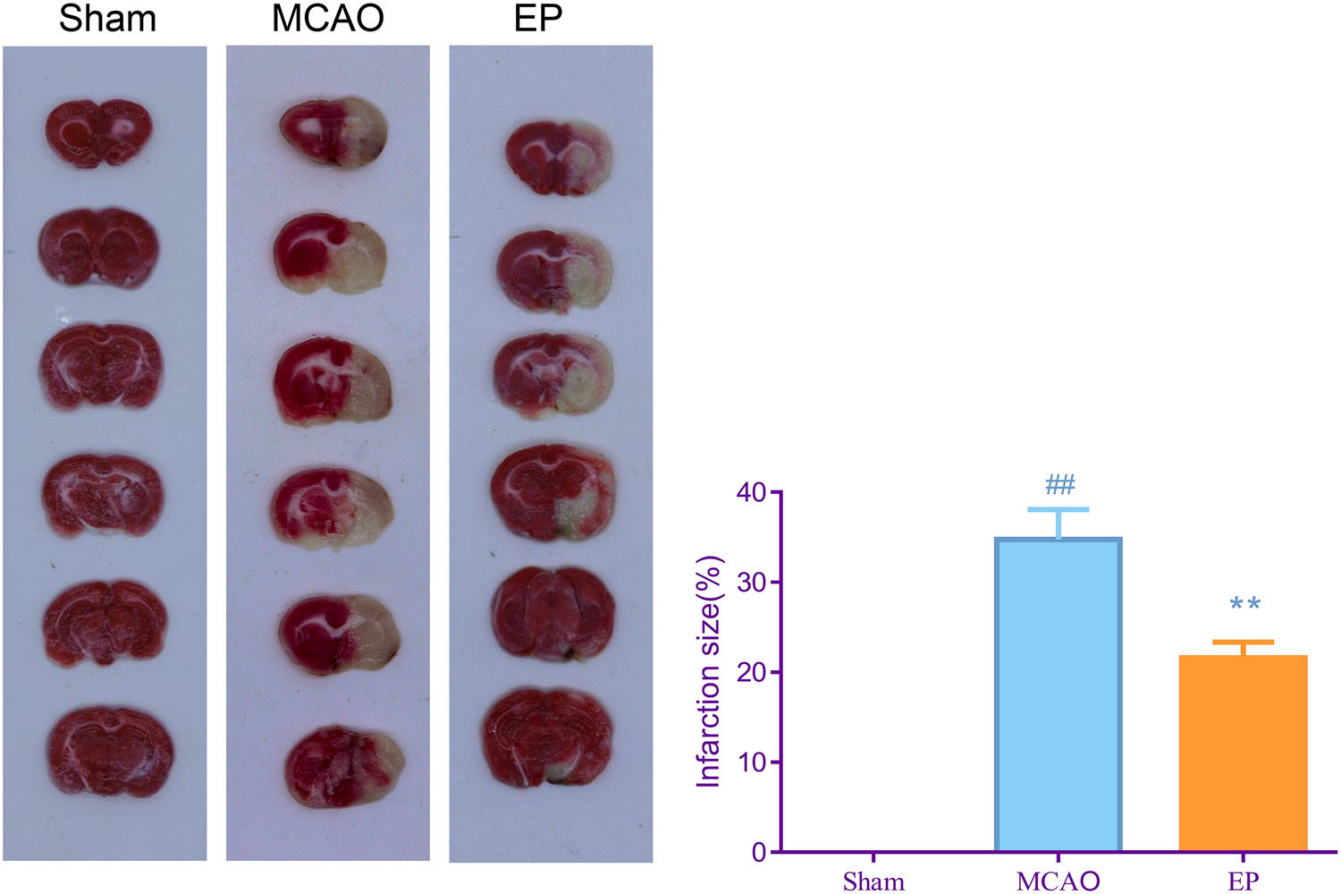
Exercise preconditioning (EP) reduced cerebral infarction for middle cerebral artery occlusion (MCAO) rats. At 48 h after cerebral ischemia reperfusion injury (CIRI), the cerebral infarct volume of the rats in each group were evaluated by TTC staining. ^#^ *p* < .05, ^##^ *p* < .01 versus Sham. *p < .05, **p < .01 versus EP. Results were presented as mean ± SD. *n* = 3. TTC, 2,3,5‐triphenyl tetrazolium chloride.

To evaluate the impact of EP on cerebral infarct volumes, TTC staining was performed in the study. Cerebral infarct volume is widely accepted as a reliable standard for measuring brain ischemic damage in animal research. The results of Figure 3 reveal that the cerebral infarction size in the MCAO group was bigger than that of the Sham group; however, EP effectively reduced the cerebral infarction size in MCAO rats (*p* < .01).

### 3.4 EP‐inhibited apoptosis of the brain in MCAO rats

**FIGURE 4:**
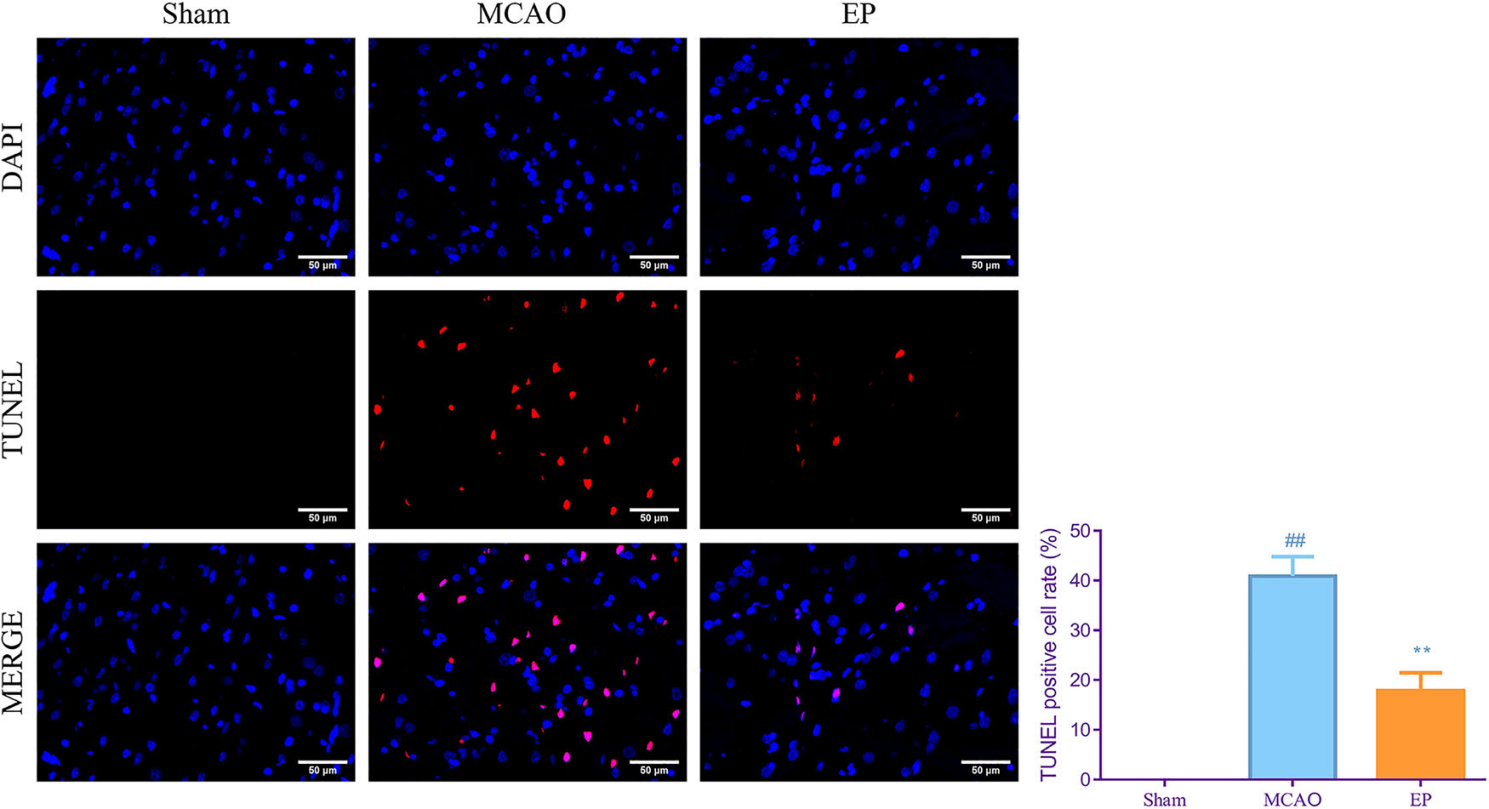
Exercise preconditioning (EP) inhibited apoptosis of cerebral cortices in middle cerebral artery occlusion (MCAO) rats. At 48 h after cerebral ischemia reperfusion injury (CIRI), the apoptosis of cerebral cortices in each group were evaluated by TUNEL (magnification, 200×). ^#^ *p* < .05, ^##^ *p* < .01versus Sham. *p < .05, **p < .01 versus EP. Results were presented as mean ± SD. *n* = 3. TUNEL, TdT‐mediated dUTP nick end labeling.

Next, TUNEL assay was conducted to detect the effect of EP on cerebral cortices in MCAO rats. Relative to the Sham group, MCAO surgery remarkably enhanced the TUNEL positive cell rate (*p* < .01). Nevertheless, a marked reduction in TUNEL positive cell rate was observed in the EP group (*p* < .01, Figure 4).

### 3.5 RNA sequencing analysis of cerebral cortices from Sham, MCAO, and EP rats

**FIGURE 5:**
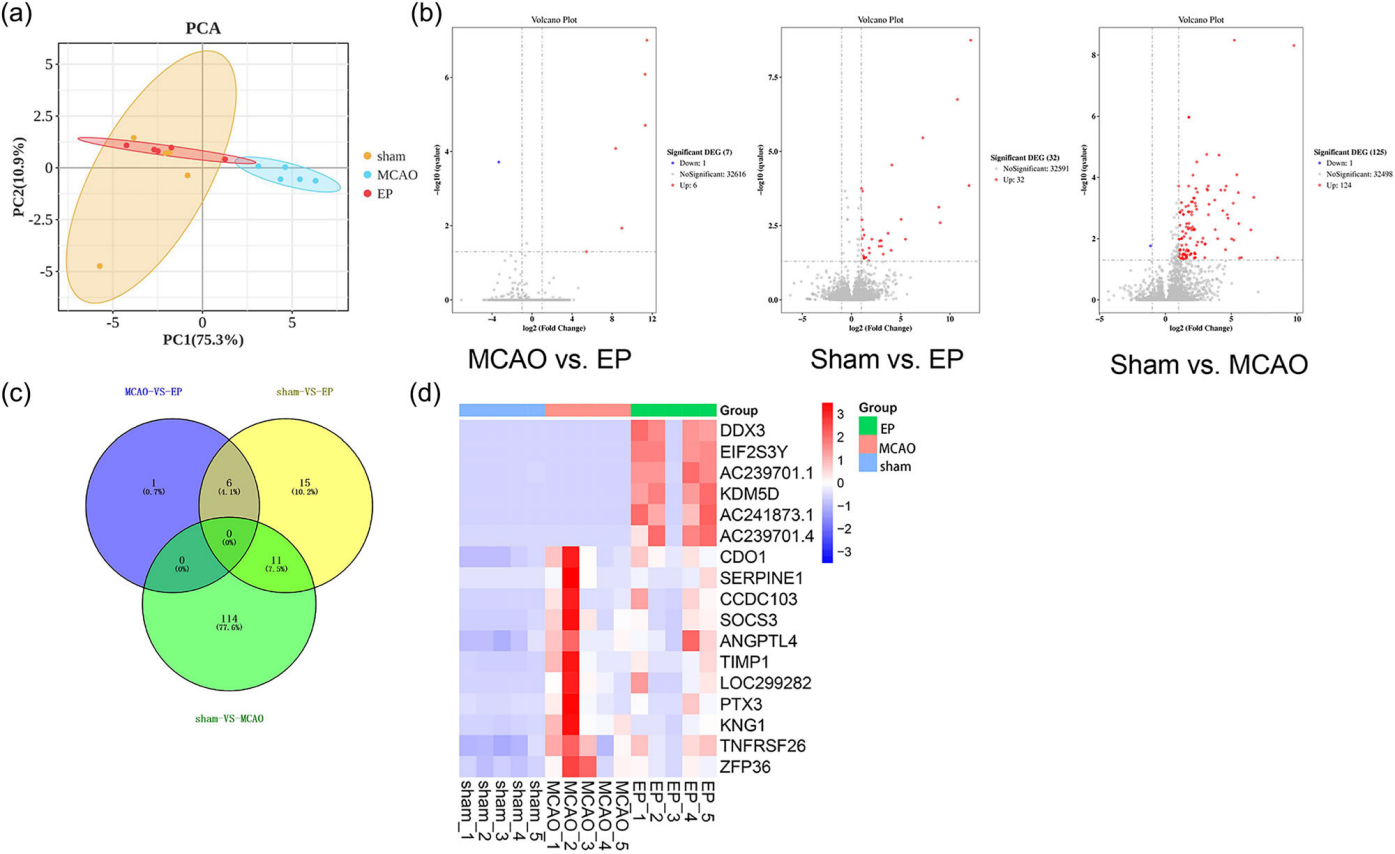
The differentially expressed genes (DEGs) identified by RNA sequencing among rats treated with or without exercise preconditioning (EP). (a) Principal component analysis. (b) Volcano plot showed significantly upregulated or downregulated DEGs among groups. (c) Venn diagram was applied to present the DEGs among group. (d) Hierarchical clustering analysis of DEGs among groups. *n* = 5. DEGs, differentially expressed genes.

To better understand the mechanism of EP in MCAO, RNA sequencing was performed on total RNA extracted from the cerebral cortices of rats in the Sham, MCAO, and EP groups. Principal component analysis results showed a significant difference in the RNA of the cerebral cortices between the Sham and MCAO groups. The RNA of the cerebral cortices from the rats in the EP group was separated from the MCAO group and closer to the Sham group (Figure 5a). Then, volcano maps (Figure 5b) were applied to present the DEGs among the three groups. In the MCAO versus EP, one DEG was downregulated and six DEGs were upregulated. For Sham versus EP, no DEGs were downregulated but 32 DEGs were upregulated. In the Sham versus MCAO, 1 DEG was downregulated, and 124 DEGs were upregulated. Subsequently, Venn diagrams revealed that there were 17 DEGs overlapped among the groups (Figure 5c), such as TIMP1, SOCS3, ANGPTL4, CDO1, SERPINE1, and so on. The complete information of the 17 DEGs could be found in Table 2. Hierarchical clustering analysis revealed the mRNA expression profile of these 17 DEGs among the Sham, MCAO, and EP groups, the color in Figure 5d represented the levels of gene expressions in each sample (red for high level, blue for low level).

**TABLE 2:** The complete information about the 17 differentially expressed genes.

Groups	Gene symbol
MCAO vs. EP and Sham vs. EP	DDX3
	EIF2S3Y
	AC239701.1
	KDM5D
	AC241873.1
	AC239701.4
Sham vs. EP and Sham vs. MCAO	CDO1
	SERPINE1
	CCDC103
	SOCS3
	ANGPTL4
	TIMP1
	LOC299282
	PTX3
	KNG1
	TNFRSF26
	ZFP36

**FIGURE 6:**
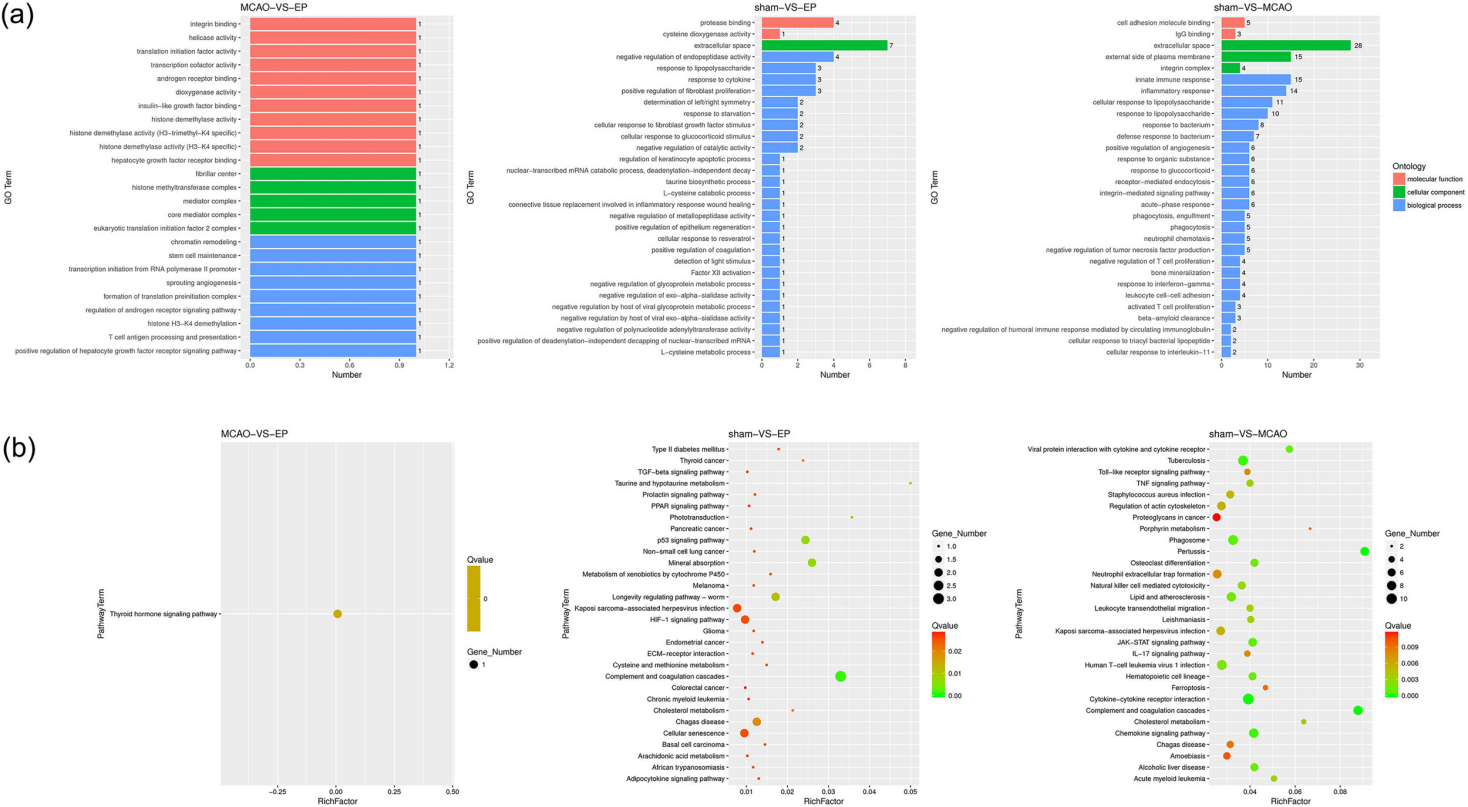
Functional enrichment analysis of exercise preconditioning (EP) in middle cerebral artery occlusion (MCAO) rats. (a) Results of GO analysis. (b) Results of KEGG analysis. GO, Gene ontology; KEGG, kyoto encyclopedia of genes and genomes.

Next, GO analysis was conducted on all DEGs, and the results found most of the DEGs were related to extracellular space, negative regulation of endopeptidase activity, protease binding, and so on (Figure 6a). Moreover, we also performed KEGG enrichment analysis to further identify DEGs‐enriched pathways among the rats in the Sham, MCAO, and EP groups. The results of Figure 6b reveal that most DEGs were enriched in signal pathways such as the HIF‐1 signaling pathway, Kaposi sarcoma‐associated herpesvirus infection, cellular senescence, proteoglycans in cancer, and so on.

### 3.6 EP suppressed TIMP1, SOCS3, ANGPTL4, CDO1, and SERPINE1 expressions in MCAO rats

**FIGURE 7:**
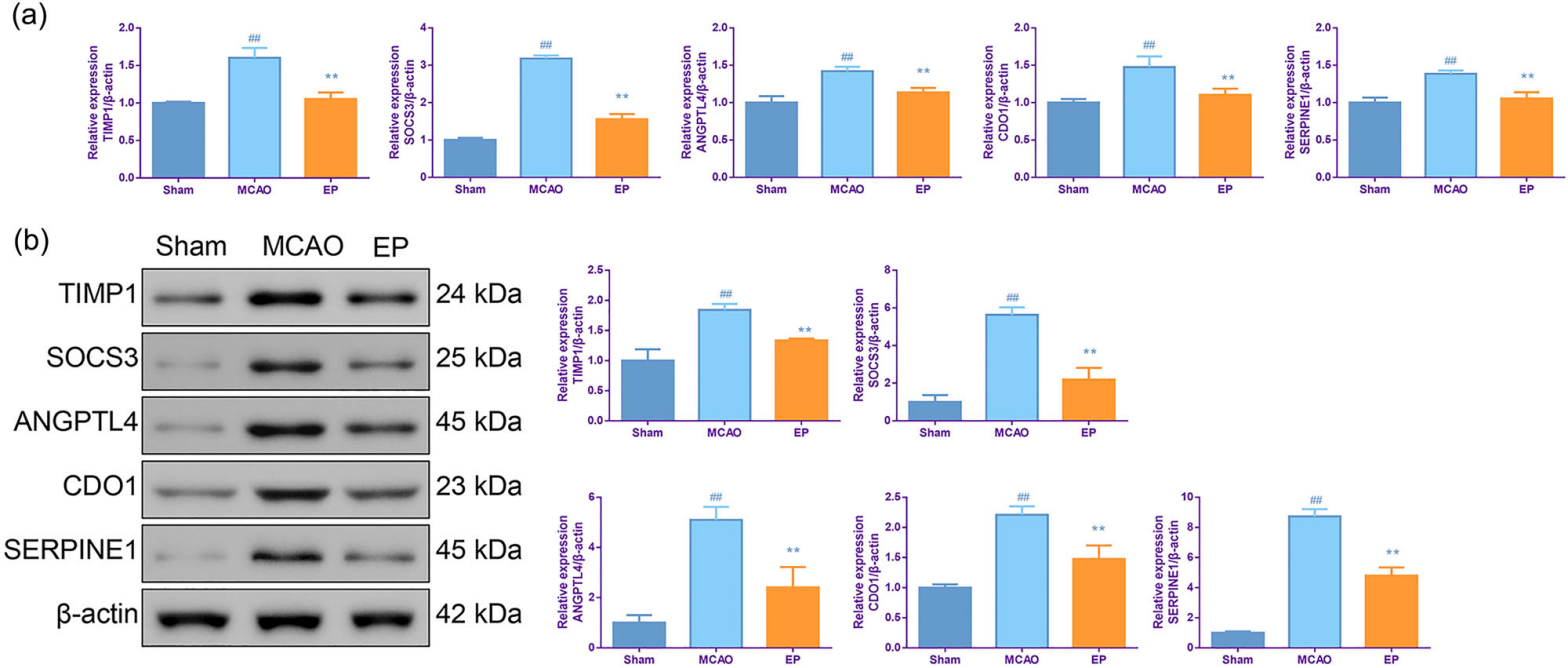
Exercise preconditioning (EP) suppressed TIMP1, SOCS3, ANGPTL4, CDO1, and SERPINE1 expressions of the cerebral cortices in middle cerebral artery occlusion (MCAO) rats. At 48 h after cerebral ischemia reperfusion injury (CIRI), TIMP1, SOCS3, ANGPTL4, CDO1, and SERPINE1 expression levels of cerebral cortices in each group were evaluated by qPCR (a) and Western blotting (b). ^#^ *p* < .05, ^##^ *p* < .01 versus Sham. *p < .05, **p < .01 versus EP. Results were presented as mean ± SD. *n* = 3.

Next, the expressions of some key DEGs in the cerebral cortical tissues were further verified by qPCR and Western blotting analysis. As illustrated in Figure 7a, the mRNA levels of TIMP1, SOCS3, ANGPTL4, CDO1, and SERPINE1 in the cerebral cortical tissues were significantly upregulated in the MCAO group (*p* < .01). However, EP obviously downregulated the expressions of these mRNA (*p* < .01). The results of Western blotting analysis were similar to the results of qPCR (Figure 7b).

## 4 DISCUSSION

CIRI is a common and severe clinical condition with limited treatment options (Lin et al., 2020). MCAO surgery is widely considered a classical method to mimic CIRI in humans, which exhibits a high consistency with the pathological alterations observed in CIRI patients; moreover, MCAO also has the strengths of short operative time and leading to minimal harm to animals (Chen et al., 2018; Jiang et al., 2023). As the MCAO‐induced animal model exhibited similar symptoms to CIRI patients as well as the strengths of high repeatability and good stability, MCAO‐induced animal model is frequently used to explore the function and mechanism of drugs in CIRI (Fu et al., 2021; Huang et al., 2021; Jun et al., 2023). Thus, MCAO rat model was adopted in the study. In the study, we found the MCAO rats exhibited higher neurological deficit scores, more dead neurons, larger cerebral infarction size as well as more apoptosis cells in cerebral cortical tissues, which demonstrated the success of the CIRI model establishment.

Recent studies have suggested that EP can exert neuroprotective functions through multitarget, multichannel, and multilevel mechanisms. Published studies have shown that EP is able to alleviate blood–brain barrier (BBB) function, mitigate ischemic brain damage, enhance brain blood flow, modulate endothelin‐1 expression, and ameliorate neurological deficits following CI via regulating TIMP1 and MMP9 expression imbalances (Zhu et al., 2016). Additionally, EP has also been reported to alleviate memory impairment following CI by protecting hippocampal neurons from ischemia‐caused degeneration (Shamsaei et al., 2015). Furthermore, Shan et al. (2023) have found that through suppressing apoptosis and inflammation, EP can alleviate neurological deficits and reduce infarct volume. Consistently, our study found that EP reduced neurological deficit scores, neuron mortality, cerebral infarction size, and inhibited apoptosis in cerebral cortical tissues for rats underwent MCAO, which indicated that EP had the potential for CIRI treatment.

RNA sequencing is proving to be an effective tool in investigating the molecular mechanisms of drugs on diseases (Wang, Wang et al., 2021). In the present study, we explored RNA sequencing to uncover the potential mechanisms by which EP can mitigate MCAO. The results revealed that there were 17 DEGs, such as TIMP1, SOCS3, ANGPTL4, CDO1, and SERPINE1, that were closely associated with the therapeutic effect of EP to MCAO. Furthermore, our KEGG analysis indicated that these DEGs were mainly enriched in the HIF‐1 signaling pathway, Kaposi sarcoma‐associated herpesvirus infection, cellular senescence, proteoglycans in cancer, and so on. Based on the KEGG results, HIF‐1 pathway, the pathway associated with the disease and enriched with 2 DEGs (TIMP1 and SERPINE1) was further selected for our future study. In addition, studies have indicated that TIMP1, SOCS3, ANGPTL4, CDO1, and SERPINE1 are closely associated with CIRI (Liang et al., 2021; Nie et al., 2022; Pu et al., 2022; Wang, Lin et al., 2021) and the HIF‐1 pathway (Cui et al., 2015; Fujii et al., 2020; Li et al., 2021; Qi et al., 2023; Warnhoff et al., 2024). Consequently, the expressions of these genes in cerebral cortical tissues were analyzed at both the mRNA and protein levels, and the results of qPCR and Western blotting revealed that EP downregulated TIMP1, SOCS3, ANGPTL4, CDO1, and SERPINE1 expressions in MCAO rats.

Among these DEGs, it was believed that TIMPs should be considered vital genes to involve the onset and progression of CIRI (Chang et al., 2015). TIMP1 is an effective inhibitor for MMPs. Its primary function is to block the binding of matrix metalloproteinases (MMPs) to the substrate by combining the cysteine residue in the amino acid functional area with the zinc ion active center of activated MMPs, thereby preventing the degradation of the extracellular matrix (Maddahi et al., 2009). Histological analysis of human specimens provides evidence that TIMP1 concentration in infarcted brain tissues is higher than in healthy brain tissues (Cuadrado et al., 2009). Intervening TIMP1 expression could serve as a potential strategy for the prevention and treatment of CIRI. Notably, one study has found that exercise can attenuate pulmonary disease by regulating TIMP1 expression (Toledo et al., 2012). Another research also has demonstrated that exercise training can regulate the concentration of TIMP‐1 in diabetic cardiomyopathy mice, thereby improving cardiac function (Dede et al., 2022). Therefore, we hypothesized that EP may alleviate CIRI via the regulation of TIMP1.

HIF‐1α is a critical nuclear transcription factor under ischemia and hypoxia in mammals (Carrasco‐Pozo et al., 2020). Substantial evidence has demonstrated that the HIF‐1 pathway is involved in CIRI pathogenesis, and mediating the HIF‐1 pathway can provide neuroprotection against CIRI (Yuan et al., 2011). Under hypoxia, the expression of HIF‐1α in neuronal cells was upregulated as suppressed degradation. The accumulated HIF‐1α subsequently translocated to the nucleus and bound to the hypoxia response element sequence of the target gene promoter, eventually disrupting BBB integrity and contributing to neuronal death (Hou et al., 2019). In addition, the HIF‐1 pathway is also strongly related to senescence (Liu, Huang et al., 2021), which is the primary independent risk factor for stroke occurrence and unfavorable outcomes (Zhou et al., 2017). Animal research has indicated that the HIF‐1α pathway is activated in the brain tissues of CIRI rats (Zhang et al., 2012); however, inhibiting the HIF‐1α pathway can improve BBB integrity of CIRI animals (Long et al., 2023). Wang et al. (2013) have reported that EP may have a protective effect on ischemic brains through the regulation of HIF‐1α. In addition, exercise may have a cardioprotective function by suppressing the HIF‐1α pathway (Arabzadeh et al., 2022). Therefore, we hypothesized that regulation of the HIF‐1α pathway may be one of the main mechanisms by which EP ameliorates MCAO.

However, we did not perform experiments to validate whether EP alleviates CIRI via regulation of TIMP1 expression and HIF‐1α pathway, which is the major limitation of our study. In the future, we will interfere with TIMP1 expression and HIF‐1α pathway to further elucidate the exact mechanism of EP on MCAO. Furthermore, the downregulation effects of EP on SOCS3, ANGPTL4, CDO1, and SERPINE1 expressions in MCAO rats were also significant. It would be beneficial to further verify whether SOCS3, ANGPTL4, CDO1, and SERPINE1 expressions will be regulated by TIMP1 in MCAO rats.

In conclusion, this study suggested that EP could improve neurological deficit scores, alleviate neuronal death of the brain, attenuate cerebral infarct volume as well as inhibit brain cell apoptosis for MCAO rats. These effects may be attributed to the regulation of TIMP1 expression and the HIF‐1α pathway. All these indicated that EP appears to be beneficial for patients who are at risk of CIRI.

## AUTHOR CONTRIBUTIONS

**Yan Wu**: Writing—original draft. **Hui Yang**: Formal analysis. **Feifeng Chen**: Data curation. **Baohua Li**: Formal analysis; data curation. **Xiangbo Meng**: Conceptualization; funding acquisition.

## CONFLICT OF INTEREST STATEMENT

The authors declare no conflicts of interest.

### PEER REVIEW

The peer review history for this article is available at https://publons.com/publon/10.1002/brb3.3608

## Data Availability

The datasets generated during and/or analyzed during the current study are available from the corresponding author upon reasonable request.
